# Calib-Net: Calibrating the Low-Cost IMU via Deep Convolutional Neural Network

**DOI:** 10.3389/frobt.2021.772583

**Published:** 2022-01-03

**Authors:** Ruihao Li, Chunlian Fu, Wei Yi, Xiaodong Yi

**Affiliations:** ^1^ Artificial Intelligence Research Center (AIRC), Defense Innovation Institute, Beijing, China; ^2^ Tianjin Artificial Intelligence Innovation Center (TAIIC), Tianjin, China

**Keywords:** IMU calibration, deep neural network, orientation estimation, spatio-temporal, visual inertial odometry

## Abstract

The low-cost Inertial Measurement Unit (IMU) can provide orientation information and is widely used in our daily life. However, IMUs with bad calibration will provide inaccurate angular velocity and lead to rapid drift of integral orientation in a short time. In this paper, we present the Calib-Net which can achieve the accurate calibration of low-cost IMU via a simple deep convolutional neural network. Following a carefully designed mathematical calibration model, Calib-Net can output compensation components for gyroscope measurements dynamically. Dilation convolution is adopted in Calib-Net for spatio-temporal feature extraction of IMU measurements. We evaluate our proposed system on public datasets quantitively and qualitatively. The experimental results demonstrate that our Calib-Net achieves better calibration performance than other methods, what is more, and the estimated orientation with our Calib-Net is even comparable with the results from visual inertial odometry (VIO) systems.

## 1 Introduction

Low-cost inertial measurement units (IMU) are widely used in our daily life (Nebot and Durrant-Whyte, 1999; Ahmad et al., 2013; Rehder and Siegwart, 2017). Cheap IMU can provide the attitude and position information by integrating three-axis angular velocity measurements and three-axis linear acceleration measurements. Many devices (such as smartphones, autonomous robots, AR/VR devices, etc) are equipped with low-cost IMU sensors to obtain inertial measurements in order to achieve different features. However, the accuracy of IMU is easily affected by calibration parameters include scaling factors, axes misalignments, etc. Inaccurate calibration will produce imprecise angular velocity and linear acceleration, and the integration operation will lead to rapid error accumulation and estimation drift.

In order to limit the drift as much as possible, precise IMU calibration must be performed for both inertial odometry (IO) and visual-inertial odometry (VIO). A lot of research about IMU calibration or camera/IMU calibration are studied in the past several years. Most methods (Furgale et al., 2013; Rohac et al., 2015; Rehder et al., 2016) prefer to design a mathematical model and take calibration model parameters as constant values. External devices are usually needed for the pre-calibration procedure. Online calibration methods (Qin and Shen, 2018; Yang et al., 2020) which optimize the spatio-temporal camera/IMU model parameters all together are proved effective for the tracking performance of VIO systems. As deep learning technology has achieved great success in the past decade, many researchers also introduce deep learning technology into tasks like orientation estimation (Esfahani et al., 2019b), inertial odometry (Chen et al., 2018; Esfahani et al., 2019a; Brossard et al., 2020a), visual-inertial odometry (Clark et al., 2017; Chen et al., 2019b), and most of them adopt the recurrent neural network which is memory consuming and computing consuming.

In this paper, we propose a lightweight and efficient deep convolutional neural network for low-cost IMU calibration. It is designed based on a mathematical model and is trained driven by historical data. The proposed method can calibrate the IMU measurements dynamically. By using the calibrated IMU measurements, accurate orientation estimation can be obtained and the results can even be comparable with VIO methods.

Our contribution can be summarized as below:

• We present a deep convolutional neural network called Calib-Net for low-cost IMU calibration. The Calib-Net adopts dilation convolution for spatio-temporal feature extraction, and learns to produce the compensation for gyroscope measurements dynamically.

• We introduce a mathematical calibration model to construct the training and calibration framework. Both constant calibration matrix (3 × 3) and Calib-Net hyper-parameters can be trained and optimized driven by a carefully designed loss function.

• We implement the proposed framework and validate it quantitatively and qualitatively on public datasets. The experimental evaluations show that our Calib-Net achieves satisfactory performance in IMU calibration, and is also fruitful for VIO systems.

The rest of this paper is organized as follows. Section 2 introduces the related works of IMU calibration and orientation estimation. Section 3 introduces the overview architecture of the proposed calibration framework. Section 4 describes the details of the used mathematical model, neural network architecture, and the loss function. Section 5 presents the results of experimental evaluations. Finally, the conclusion is drawn in Section 6.

## 2 Related Work

In the late 1990s, Nebot and Durrant-Whyte (1999) presented an inertial error model for gyros and accelerometers aiming at the IMU calibration for vehicle applications. Tedaldi et al. (2014) proposed an IMU calibration method without using external equipments, they need to move the IMU device by hand and place it in multiple static positions. Based on a specific sensor error model, Rohac et al. (2015) performed the low-cost inertial sensor calibration to obtain the model parameters using the nonlinear optimization under static conditions. Furgale et al. (2013) present the open-source Kalibr toolbox for the spatial and temporal calibration of multiple sensors (cameras/IMUs). Afterward, Rehder et al. (2016) extended the Kalibr toolbox and enabled precise IMU intrinsic calibration following an inertial noise model. They Rehder and Siegwart (2017) then further improved the calibration method by introducing the displacement of individual accelerometer axes into the mathematical noise model. Barrau and Bonnabel (2020) recently adopted Lie group for IMU error propagation. These calibration methods usually adopt different inertial model variants and estimate the parameters of these sensor error models. For the calibration methods above, the parameters (scale factors, misalignment errors, offsets, etc.) are usually taken as constant values. Nevertheless, these parameters are always varying as time goes on and the environment changes.

Aiming at the visual-inertial navigation system, Qin and Shen (2018) presented an online calibration method to optimize model parameters dynamically, and proposed VINS-mono system (Qin et al., 2018) which is one of the state-of-the-art visual-inertial systems. Yang et al. (2020) studied and discussed the necessity and importance of online IMU intrinsic calibration for visual-inertial navigation systems, and proved that online calibration is helpful for improving fusion performance. They also demonstrated an open-source visual-inertial odometry system called OpenVINS (Geneva et al., 2020). Campos et al. took the IMU measurement uncertainty into account (Campos et al., 2020), and proposed to use maximum a posteriori (MAP) estimation during IMU initialization in the ORB-SLAM3 system (Campos et al., 2021). Most of these methods rely on a mathematical model and need to calibrate every device before use.

As to the usage of deep learning in IMU calibration and propagation, Yan et al. (2018) proposed to use the machine learning technology to regress velocity vector with linear accelerations and angular velocities as inputs. Nobre and Heckman (2019) proposed to model the IMU calibration as a Markov Decision Process (MDP) and use reinforcement learning to achieve the regression of calibration parameters. Clark et al. (2017) presented VINet which takes the visual-inertial odometry problem as a sequence-to-sequence learning problem to solve and avoids manual camera/IMU calibration operation. Chen et al. (2018) introduced IONet for inertial odometry using the recurrent neural network. Ionet formulates the odometry as an optimization problem based on the neural network and estimates the trajectories with raw measurements as network input. Afterward, they Chen et al. (2019a) further extended the network and also used it to predict model uncertainty. However, the above learning methods usually adopt neural networks with large weights which will consume lots of computation resources.

Esfahani et al. (2019b) presented a recurrent neural network called OriNet for orientation estimation. 3D orientation can be obtained by OriNet with only low-cost IMU measurements as the network input. They Esfahani et al. (2019a) also proposed AbolDeepIO which simulates the noise model during training and achieves robust inertial odometry with a novel deep neural network. Instead of using recurrent neural networks, Brossard et al. (2020b) adopted a convolutional neural network to estimate the orientation with an IMU device. They Brossard et al. (2020a) further achieved accurate and robust dead-reckoning with only a commercial IMU. The Kalman filter is introduced and a deep neural network is utilized to predict the dynamic parameters of the filter.

## 3 Methods

### 3.1 Mathematical Model for Low-Cost IMU Calibration

#### 3.2 Architecture Overview

**FIGURE 1:**
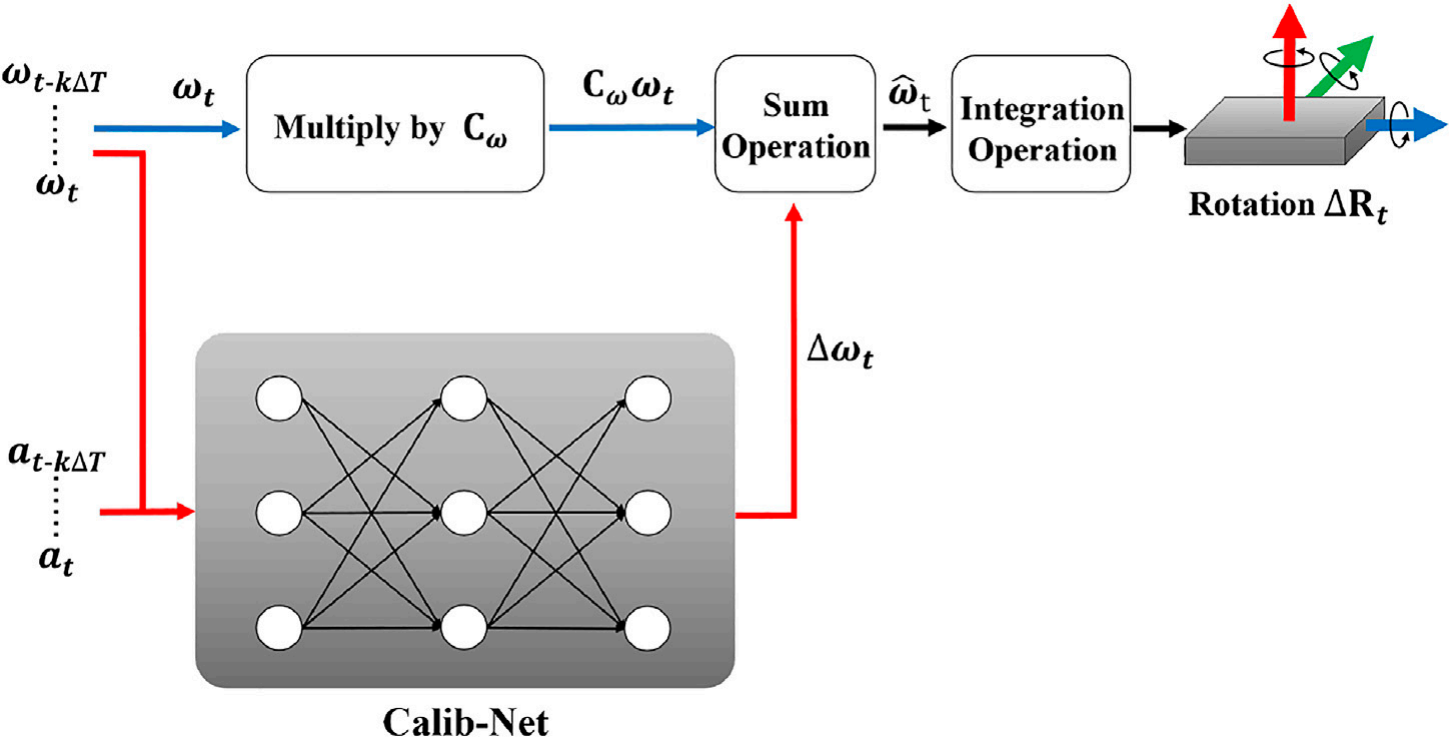
The overview of the proposed framework. By selecting a mathematical model, we introduce a simple but effective convolutional neural network for dynamic IMU calibration.

The overview of our proposed framework is shown in Figure 1. Calib-Net takes sequential gyroscope measurements and acceleration measurements as the input, and outputs the compensation for raw IMU measurements. Dilation convolution is utilized for spatio-temporal feature extraction instead of recurrent neural networks. By designing a mathematical calibration model, the measurements can be dynamically calibrated and corrected with the network output. In this way, accurate orientation can be directly obtained through simple integration. Based on only hundred seconds of labeled data, the hyperparameters of the Calib-Net and the constant model parameters (matrix includes) can be optimized at the same time driven by a carefully designed loss function.

For a typical low-cost Inertial Measurement Unit (IMU), it usually consists of a three-axis gyroscope, a three-axis accelerometer, and sometimes a magnetometer. The three-axis gyroscope can provide angular velocity information, the three-axis accelerometer can provide the linear acceleration information. Considering the noise and bias of the sensor measurements (Rehder et al., 2016), the IMU sensor model can be represented as below:

$$\left[\begin{array}{c} {\hat{\omega }}_{t}\\ \hat{a_{t}} \end{array}\right]=C\left[\begin{array}{c} \omega_{t}\\ a_{t} \end{array}\right]+\left[\begin{array}{c} b_{t}^{\omega }\\ b_{t}^{a} \end{array}\right]+\left[\begin{array}{c} n_{t}^{\omega }\\ n_{t}^{a} \end{array}\right]$$ (1)

Where angular velocity $\omega_{t}\in R^{3}$ and linear acceleration $a_{t}\in R^{3}$ are the measurements of gyroscope and accelerometer, $b_{t}^{\omega }\in R^{3}$ and $b_{t}^{a}\in R^{3}$ are the gyroscope and accelerometer bias, $n_{t}^{\omega }\in R^{3}$ and $n_{t}^{a}\in R^{3}$ are additive zero-mean Gaussian noises for gyroscope and accelerometer, angular velocity ${\hat{\omega }}_{t}\in R^{3}$ and linear acceleration $\hat{a_{t}}\in R^{3}$ are the calibrated measurements which would be used for integration. The **C** is the intrinsic calibration matrix (approximate equals to **I**_6_) for the IMU model (Rehder et al., 2016), and can be modeled as:

$$C=\left[\begin{array}{cc} C_{\omega } & C_{\times }\\ 0_{3\times 3} & C_{a} \end{array}\right]$$ (2)

Where **C**_*ω*_ contains scale factor and axis misalignment for gyroscope measurements, **C**_*a*_ contains scale factor and axis misalignment for accelerometer measurements, both **C**_*ω*_ and **C**_*a*_ are approximately equal to identity matrix **I**_3_. **C**_×_ is the coefficient matrix. It indicates the effect that the linear accelerometer has on the gyroscope, and is approximately equal to **0**_3×3_. So the gyroscope measurement (angular velocity) model can be represented as:

$${\hat{\omega }}_{t}=C_{\omega }\omega_{t}+C_{\times }a_{t}+b_{t}^{\omega }+n_{t}^{\omega }=C_{\omega }\omega_{t}+\Delta \omega_{t}$$ (3)

In the formula above, we abbreviate the compensation component for gyroscope measurements as:

$$\Delta \omega_{t}=C_{\times }a_{t}+b_{t}^{\omega }+n_{t}^{\omega }$$ (4)

Where the compensation component Δ*ω*_*t*_ is related to both gyroscope measurements and accelerometer measurements. By integrating the corrected angular velocity ${\hat{\omega }}_{t}$ , we can achieve the estimation of 3D rigid rotation follows the equation below:

$$R_{t+\Delta T}=R_{t}Exp\left({\hat{\omega }}_{t}\Delta T\right)$$ (5)

Where **R**_*t*_ ∈ *SO*(3) and **R**_*t*+Δ*T*_ ∈ *SO*(3) are the rotation matrices. *t* is the timestamp that IMU outputs measurements, and Δ*T* is the minimal time interval between two consecutive IMU readings. In our case, the IMU runs at 200 Hz, and the Δ*T* is 5 ms. Exp (⋅) is the exponential map for SO(3). As indicated in the above formula, incorrect ${\hat{\omega }}_{t}$ will lead to continuous error accumulation as more integrations propagate.

### 3.3 Calib-Net for IMU Measurement Correction

**FIGURE 2:**
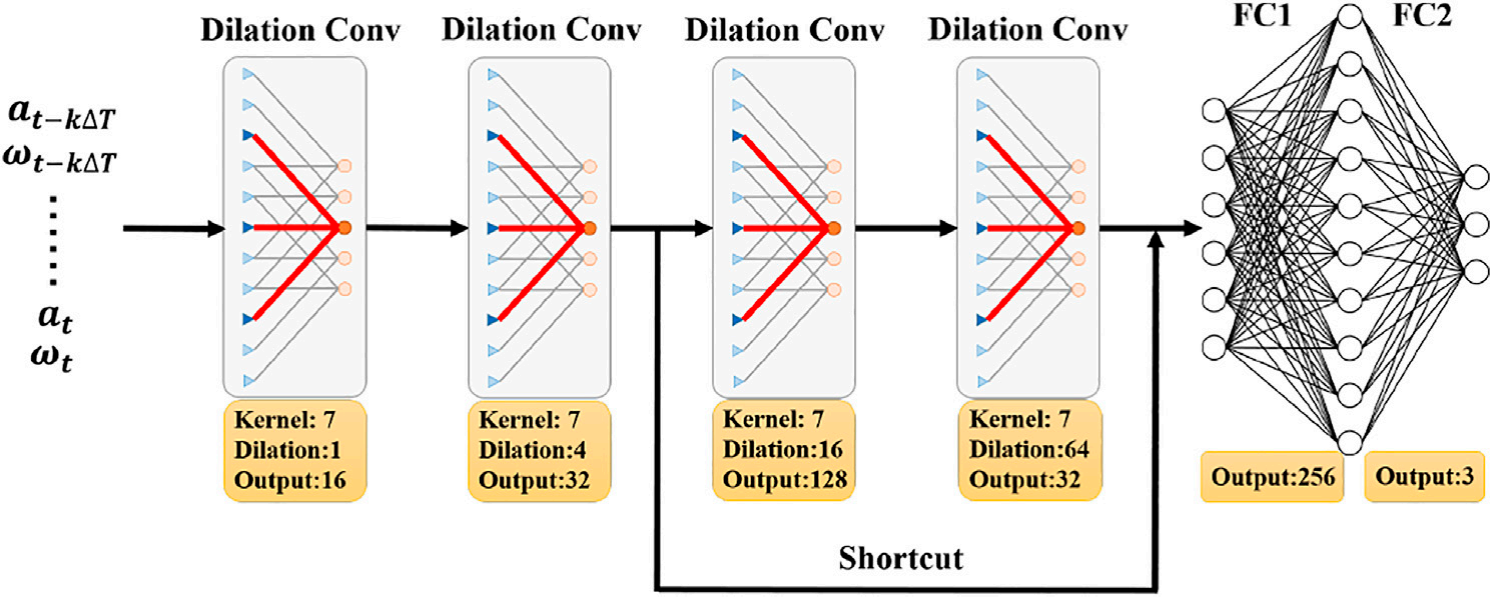
The details of the Calib-Net structure. The proposed Calib-Net takes temporal gyroscope measurements and accelerometer measurements as inputs, and outputs the compensation part for angular velocity measurements.

As shown in Eq. 3 and Figure 1, **C**_*ω*_ and Δ*ω*_*t*_ play an important role in IMU calibration and orientation estimation. In most cases, **C**_*ω*_ will be taken as a constant matrix, and Δ*ω*_*t*_ is a small time-varying vector affected by many factors. In our proposed calibration system, we use the Caib-Net to estimate the compensation part Δ*ω*_*t*_ for angular velocity measurements. The detailed structure of the Calib-Net is shown in Figure 2. It takes the long temporal sequential gyroscope measurements and accelerometer measurements as the network input, and network can be represented as below:

$$\Delta \omega_{t}=F\left(\omega_{t-k\Delta T},a_{t-k\Delta T},\ldots ,\omega_{t},a_{t}\right)$$ (6)

**FIGURE 3:**
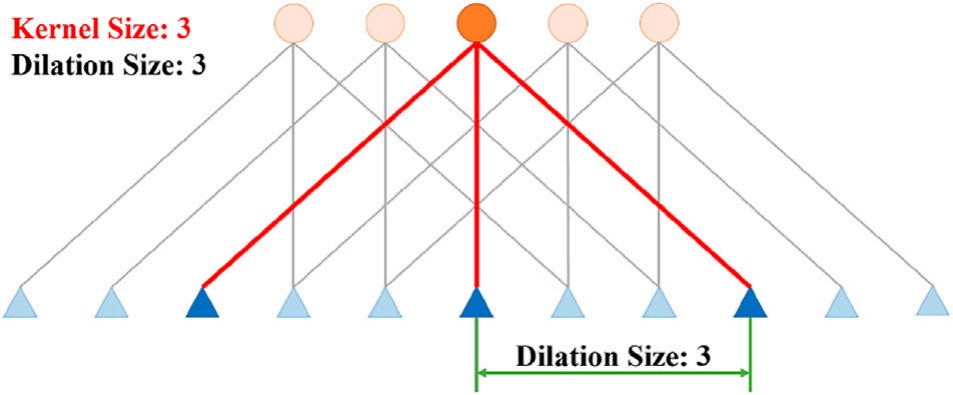
The illustration of 1D Dilation Convolution. Dilation size indicates the spacing between the convolution kernel points.

Where *k* is the number of IMU readings used as the input of the proposed Calib-Net. *F* (⋅) represents the nonlinear function that the Calib-Net stands for. Dilation convolution is adopted to extract spatio-temporal features of IMU measurements. As shown in Figure 3, by introducing the dilation convolution, the historically temporal measurements are fed into the network and used for feature extraction. Dilation size in dilation convolution indicates the spacing between the convolution kernel points, so with different kernel sizes and dilation sizes, different temporal IMU readings will be taken accordingly. To be specific, the length of the input depends on the product of kernel size and dilation size. In the presented network shown in Figure 2, the maximum product value is 448 which means the network will take *k* = 448 IMU readings as the network input.

The Calib-Net is composed of two dilation convolutional layers, one residual block, and two fully connected layers. The residual block includes two dilation convolutional layers and one shortcut. The detailed configuration of each layer is given in Figure 2. As to the matrix **C**_*ω*_, we set it to the trainable variable. In this way, following the mathematical model shown in Figure 1, the parameters of Calib-Net and Δ*ω*_*t*_ can be trained and optimized driven by the carefully designed loss function.

### 3.4 Loss Function for Regression

There are many losses that are widely used for solving the regression problem (Jadon, 2020). The losses include L1 Loss using Mean Square Error (MSE), L2 Loss using Mean Absolute Error (MAE), Huber Loss using Smooth Mean Absolute Error, etc. L1 Loss is robust to outliers. however, its gradient is always the same during the training and is still large even faced with small loss values. In this way, it is hard and inefficient for L1 Loss to find the minima at the end of training. On the contrary, L2 Loss is sensitive to outliers, but it is easier for L2 Loss to find a stable solution. Huber loss combines the advantages of L1 Loss and L2 Loss, it is more robust to outliers than L2 Loss and can decrease the gradient around the minima.

In our proposed Calib-Net, we choose to use the log hyperbolic cosine (log-cosh) loss for gyroscope measurement correction regression. Compared with the Huber Loss, it approximately equals (*y* − *y*′)^2^/2 for the small loss and to $\left|y-y^{\prime }\right|-\log\left(2\right)$ for the large loss, so it has all the advantages of Huber Loss and is twice differentiable for both small loss and large loss. The loss function is defined as below:

$$L\left(y,y^{\prime }\right)=\overset{n}{\underset{i=1}{\sum }}\log\left(\cosh\left(y-y^{\prime }\right)\right)$$ (7)

Where *y* is the label (ground truth) of orientation in our case, and *y*′ is the estimated orientation using the calibrated gyroscope value (namely angular velocity).

We also use multi-scale orientation loss to achieve better calibration performance. The orientation is estimated through the integration of calibrated angular velocity. So we adopt different time intervals to compute the loss and perform the back-propagation driven by the loss. The loss used for network training is shown below:

$$L=\overset{n}{\underset{i=1}{\sum }}L_{\Delta t}+\overset{n/2}{\underset{i=1}{\sum }}L_{2\Delta t}+\overset{n/4}{\underset{i=1}{\sum }}L_{4\Delta t}+\cdots +L_{n\Delta t}$$ (8)

Where *n* ⋅Δ*t* is the maximum time interval used for computing the loss, and its value is *n* = 2^*x*^. By carefully selecting different Δ*t* and *n* using the trial and error method, we can adopt the suitable rotation transformation combination to train the calibration parameters.

## 4 Experimental Evaluation

In this section, we evaluate the performance of our proposed Calib-Net using the public EuROC dataset Burri et al. (2016). Both quantitive and qualitative experiments are performed for comparison. We first compare the orientation estimation performance of our Calib-Net with different deep learning methods. Then we replace the gyroscope measurements used for the OpenVINS system with the calibrated angular velocity from our Calib-Net, and compare the performance with state-of-the-art VIO methods.

For the training of our Calib-Net, most computers with a normally configured GPU are enough. In our case, we use a desktop equipped with an Intel i7-8700K CPU with 3.7-GHz and an Nvidia GeForce GTX 1060 GPU with 6 GB memory. The framework of Calib-Net is implemented based on PyTorch. We use the ADAM optimizer for the network training, the learning rate is set to 0.01, and the weight decay is 0.1. The consine anneal warm restart scheduler is adopted for network learning. The weight parameter for log-cosh loss is 1e6. The IMU runs at 200 Hz, so the Δ*T* is 5 ms in our case. For loss computing, we set Δ*t* to 80 ms and *n* to 2 (as illustrated in Eq. 8) which means we use 16 and 32 IMU readings for integration. We training the network for 1,200 epochs and it only takes about 8.5min with the Nvidia GTX 1060 GPU which can be reached easily. For the test procedure, our proposed lightweight Calib-Net can easily reach real-time performance for calibration and orientation estimation (the IMU reading is 200 Hz).

### 4.1 Calibration Performance Evaluation Among Learning-Based Methods

We first evaluate the proposed Calib-Net by comparing the orientation estimation performance with other learning-based methods. Absolute Orientation Error (AOE) is adopted as the evaluation metric. We choose the evaluation tool (Zhang and Scaramuzza, 2018) to compute the metric. Except for AOE, we also adopt the yaw error as the metric as achieving the accurate yaw angle estimation is most difficult for IMU.

The public EuROC dataset (Burri et al., 2016) is used for the evaluation, and the uncalibrated ADIS16448 IMU is adopted in the dataset. We take MH_01_easy, MH_03_medium, MH_05_difficult, V1_02_medium, V2_01_easy, V2_03_difficult as the training sequences, and take MH_02_easy, MH_04_difficult, V1_01_easy, V1_03_difficult, V2_02_medium as the testing sequences. As shown in Table 1, our proposed Calib-Net outperforms the raw IMU data in terms of the orientation estimation and yaw estimation. When comparing with the state-of-the-art learning-based calibration methods, our framework defeats both OriNet (Esfahani et al., 2019b) and GyroNet (Brossard et al., 2020b) on most sequences. What is more, OriNet (Esfahani et al., 2019b) adopts the Long Short-Term Memory (LSTM) component for spatio-temporal feature extraction and needs larger GPU memory. Our framework adopts dilation convolutions and proper loss function which construct an easy (less hyperparameters) but efficient (better feature learning) neural network.

**TABLE 1:** Orientation estimation results of different learning-based methods. The networks shown in the table are all trained using same sequences of the EuROC dataset (Burri et al., 2016). Both 3D orientation and yaw estimation results are given in the table. The best results are made in bold.

Seq	Calib-net		OriNet		GyroNet		Raw IMU data	
	ori. (°)	yaw (°)	ori. (°)	yaw (°)	ori. (°)	yaw (°)	ori. (°)	yaw (°)
MH_02_easy	2.01	1.91	5.75	**0.51**	**1.39**	0.85	146	130
MH_04_difficult	**0.97**	0.41	8.85	7.27	1.40	**0.25**	130	77.9
V1_01_easy	**0.79**	**0.44**	6.36	2.09	1.13	0.49	71.3	71.2
V1_03_difficult	**1.25**	**0.35**	14.7	11.5	2.70	0.96	119	84.9
V2_02_medium	**3.82**	**1.23**	11.7	6.03	3.85	2.25	117	86.0
mean	**1.77**	**0.87**	9.46	5.48	2.10	0.96	125	89.0

• training sequences: MH_01_easy, MH_03_medium, MH_05_difficult, V1_02_medium, V2_01_easy, V2_03_difficult.

• testing sequences: MH_02_easy, MH_04_difficult, V1_01_easy, V1_03_difficult, V2_02_medium.

**FIGURE 4:**
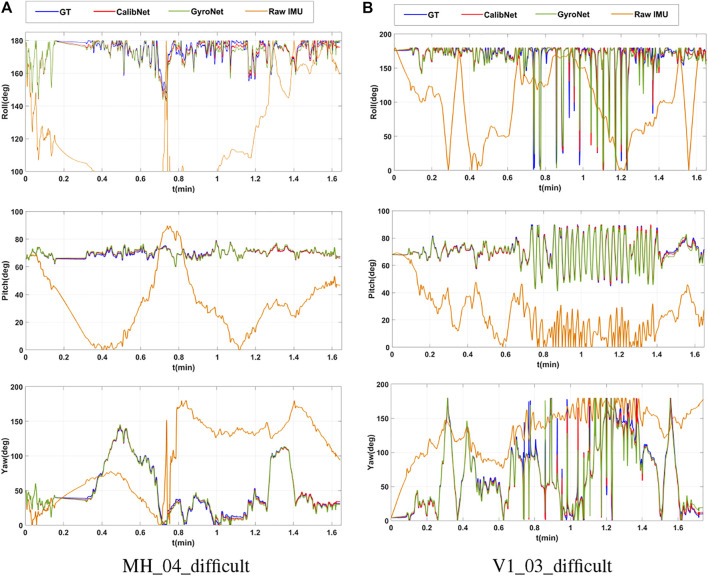
Plots of estimated orientations with different methods. **(A)** Estimated orientation for MH_04_difficult with different methods. **(B)** Estimated orientation for V1_03_difficult with different learning-based methods.

We also plot the estimated orientation in Figure 4 to give a more compact demonstration of our system’s performance. The blue line indicates the ground truth of the orientation (roll, yaw, pitch), the red line indicates the estimated orientation with our Calib-Net, the green line indicates the estimated orientation with the GyroNet, and the yellow line indicates estimated orientation from the raw IMU data. All orientations here are got only through the integration of the angular velocity. As shown in the figure, when using the raw IMU data to perform orientation estimation, the errors will be accumulated and the integrated orientation will drift in a short time due to inaccurate calibration. Our Calib-Net (red line) is most close to the ground truth and achieves the best performance among these methods.

### 4.2 Calibration Performance Evaluation Using VIO Methods

**FIGURE 5:**
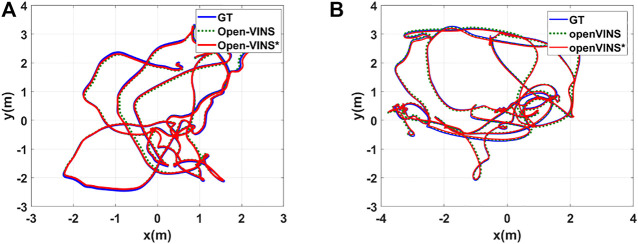
Plots of estimated trajectories with different VIO methods. **(A)** Estimated trajectories for sequence V1_01_easy. **(B)** Estimated trajectories for sequence V2_02_medium. Open-VINS* takes the corrected IMU measurements from our proposed Calib-Net.

In order to further prove the effectiveness of our proposed framework, we also introduce the calibrated angular velocity from our Calib-Net into a well-known VIO system (Open-VINS) which achieves state-of-the-art performance recently. The new VIO system combined with our Calib-Net is called OpenVINS*. We present the quantitative orientation estimation results in Table 2. As shown in the table, OpenVINS* performs better than OpenVINS in most cases, and achieves the best mean results in terms of orientation and yaw estimation. We also plot the trajectories with these methods in Figure 5. The OpenVINS* with carefully calibrated angular velocity achieves better performance in trajectory estimation. This proves that our proposed Calib-Net can accurately correct the angular velocity measurements and the calibrated angular velocity is fruitful for VIO systems.

**TABLE 2:** Orientation estimation results of different VIO methods. The Open-VINS* method takes the calibrated gyroscope data (produced by our proposed Calib-Net) as the input. Both 3D orientation and yaw estimation results are given in the table. The best results are made in bold.

Seq	Open-VINS*		Open-VINS		VINS-mono	
	ori. (°)	yaw (°)	ori. (°)	yaw (°)	ori. (°)	yaw (°)
MH_02_easy	1.40	**1.03**	**1.11**	1.05	1.34	1.32
MH_04_difficult	1.81	**1.10**	1.60	1.16	**1.44**	1.40
V1_01_easy	**0.62**	**0.42**	0.80	0.67	0.97	0.90
V1_03_difficult	2.48	2.39	**2.32**	**2.27**	4.72	4.68
V2_02_medium	**1.08**	**0.63**	1.85	1.61	2.58	2.41
mean	**1.47**	**1.11**	1.55	1.37	2.21	2.14

• training sequences: MH_01_easy, MH_03_medium, MH_05_difficult, V1_02_medium, V2_01_easy, V2_03_difficult.

• testing sequences: MH_02_easy, MH_04_difficult, V1_01_easy, V1_03_difficult, V2_02_medium.

When comparing the estimated orientation of Calib-Net (shown in table I) with that of VIO methods (shown in table II), we can find that our Calib-Net can even compete with VIO methods in terms of orientation estimation. What is more, the average yaw error of our Calib-Net is 0.87°, and it performs better than all VIO methods which include Open-VINS* with 1.11°, Open-VINS with 1.37°, VINS-Mono with 2.14°. This further indicates that a carefully calibrated low-cost IMU can achieve similar (even better in some aspects) performance when comparing with visual-inertial methods.

## 5 Conclusion and Future Work

In this paper, we present a light-weight deep convolutional neural network for low-cost IMU calibration which is called Calib-Net. A mathematical calibration model is introduced to design the training and calibration framework. Dilation convolution is adopted for spatio-temporal feature extraction of IMU measurements. Driven by a carefully designed loss function, the Calib-Net can be optimized to output the compensation for raw gyroscope measurements. Corresponding experimental evaluations are performed to prove the effectiveness of our proposed Calib-Net. The results show that our framework achieves quite good calibration performance and the orientation estimation performance of our Calib-Net can even compete with state-of-the-art VIO methods. However, the generalization ability of the proposed network to different types of IMU is still challenging, and we would like to try more datasets and study more about it. What is more, we plan to use the deep neural network to perform odometry to dead-reckon both translation and rotation. We also consider introducing deep learning technology for visual-inertial odometry.

## Data Availability

Publicly available datasets were analyzed in this study. This data can be found here: https://projects.asl.ethz.ch/datasets/.
